# Using nicotine replacement therapy for smoking reduction in pregnancy: a qualitative study of pregnant women in the UK who smoke

**DOI:** 10.1136/bmjopen-2024-085945

**Published:** 2024-08-30

**Authors:** Ross Thomson, Lucy Phillips, Sophie Orton, Felix Naughton, Tim Coleman

**Affiliations:** 1 Lifespan and Population Health, University of Nottingham School of Medicine, Nottingham, UK; 2 School of Health Sciences, University of East Anglia Faculty of Medicine and Health Sciences, Norwich, Norfolk, UK

**Keywords:** pregnant women, primary care, behavior, public health, qualitative research

## Abstract

**Abstract:**

**Objectives:**

To explore the acceptability and perceived motivations and barriers of using nicotine replacement therapy (NRT) to reduce the number of daily cigarettes smoked in pregnancy, rather than for stopping completely.

**Design:**

Telephone, semi-structured interviews, audio-recorded and transcribed verbatim. Transcripts were analysed using an inductive thematic analysis.

**Participants:**

Eighteen pregnant women in the UK, who were smoking or had recently stopped smoking, were recruited.

**Results:**

Half of interviewees reported having used NRT to reduce smoking during their current pregnancy, and there was overwhelming support for the UK National Health Service to recognise this as a potentially useful way to use these products. The cost and stigma associated with purchasing NRT products when pregnant were seen as barriers to using NRT in this way. The early offer of NRT for reduction along with a tailored, structured approach to support was seen as important.

**Conclusions:**

Using NRT to help women, who are unable to stop smoking, to reduce their smoking may be acceptable to pregnant women. This study found women were already using NRT alongside ad hoc strategies to reduce their smoking. Further research evaluating structured smoking reduction support, alongside concurrent NRT use is needed.

**STRENGTHS AND LIMITATIONS OF THIS STUDY**

- This study used online recruitment using Facebook adverts and social media posts that allowed researchers to identify and interview women from across the UK.
- The use of social media recruitment will have excluded those without internet access or who use Facebook as a social media platform.
- Using telephone, rather than face-to-face interviews, while more difficult to develop rapport with interviewees, is known to have advantages when discussing topics of a potentially sensitive nature.

## Background

 Smoking in pregnancy is a major public health problem; it is the biggest preventable cause of adverse pregnancy and perinatal outcomes.^1^,^3^ Globally, large numbers of pregnant women smoke and while slowly declining in high-income countries, rates are highest in Europe (8.1%) and the USA (5.9%).^4^ In England, in 2020/2021, 9.5% of women were smoking during childbirth, with rates highest in economically deprived areas (Blackpool 21.4%).^5^ However, an estimated 23.3% of women in the UK smoked *at some point* during pregnancy,^4^ resulting in approximately 160 824 fetuses being exposed to smoking in pregnancy annually,^6 7^ causing up to 5000 miscarriages, 300 perinatal deaths and 2200 premature births in the UK.^8^

In Europe, for non-pregnant smokers, nicotine replacement therapy (NRT) products are licenced for reduction as well as cessation of smoking,^9^ and evidence suggests that NRT used to cut down can induce successful quit attempts resulting in stopping smoking (RR for stopping smoking after using NRT to cut down, 1.87, 95% CI 1.43 to 2.44).^10^ However, following the WHO’s recommendation that there is no safe level of smoking in pregnancy,^11^ most countries’ guidelines urge abrupt cessation of smoking in pregnancy and jurisdictions, which recommend using NRT in pregnancy, only do so for cessation attempts.^12^ In the UK, the National Health Service (NHS) only offers NRT to pregnant women if they are ready to quit smoking and offers no alternative support to the 45% of women who smoke during pregnancy, but who do not make quit attempts.^13 14^ However, there is strong evidence that when pregnant women cannot achieve abstinence, reducing smoking is very likely to be better for theirs’ and their babies’ health than ‘smoking as usual’. There are dose-dependent associations between heaviness of smoking and birthweight,^15^ low birth weight,^15^,^17^ increased risks of adverse pregnancy and adverse neonatal outcomes^18^ and babies born to women smoking fewer than 10 cigarettes daily are heavier than babies born to women smoking >10 cigarettes daily.^15^ Helping pregnant women who cannot stop to instead reduce their smoking would substantially improve the health of up to 72 370 UK fetuses annually.^6 7 19^

Pregnant women are not recommended NRT for reducing smoking due to safety concerns. Many animal studies demonstrate that nicotine could be harmful to the developing fetus,^20^ and, in the USA and Australia, nicotine is classified as potentially a risk for use in pregnancy.^21^ While it would not be logical to advocate nicotine use in pregnant women who do not smoke, systematic reviews suggest using NRT instead of smoking is protective not harmful to the fetus,^22 23^ and pregnant women are exposed to far less cotinine (primary nicotine metabolite) from NRT than when smoking.^24^ Compared with when only smoking, pregnant women on NRT patches who also used cigarettes smoked less each week, exhaled less CO but had similar cotinine concentrations.^25^ Those offered ‘dual’ NRT for quitting (ie, patch and fast-acting NRT [eg, lozenge, spray, etc]) but who did not stop smoking and reported some cigarette use, smoked fewer each day, exhaled less CO and had lower saliva cotinine concentrations than when smoking only.^26^

Qualitative work suggests that some pregnant women who are trying to stop smoking already use NRT to reduce their smoking to assist this.^27^ However, other women are anxious about potential fetal harm from smoking and using NRT together, and some have reported viewing not quitting as a ‘failure’.^28^ Previous studies, however, have only reported the views of women who use NRT to help them stop smoking. We know little about the acceptability of offering NRT to pregnant women who feel unable to stop smoking to help them cut down their daily smoking instead. As this would be a substantial change to current clinical practice, if it were to be considered as a treatment option, it would be very important to fully understand women’s views on this use of NRT.

We conducted a qualitative exploration of the acceptability of pregnant women, who were not necessarily receiving stop smoking support (SSS), of using NRT in pregnancy to reduce the number of daily cigarettes smoked, and the barriers to and facilitators for them using NRT in this way, rather than for stopping smoking completely.

## Methods

We conducted a qualitative study using semi-structured telephone interviews. Ethical approval was granted by the Faculty of Medicine and Health Science Research Ethics Committee, University of Nottingham (reference number FMHS 442–0122). This paper follows the consolidated criteria for reporting qualitative research checklist for reporting qualitative research^29^

### Inclusion criteria

We included women who were aged ≥16, living in the UK, and who self-reported being currently pregnant and smoking or having quit smoking cigarettes during pregnancy. We also included women who either combined or replaced cigarette smoking during this pregnancy with other nicotine-containing products. Those who were unable to understand the study procedure sufficiently to provide consent and were unable to read or understand the study procedures in English or participate in an interview in English were excluded.

### Recruitment

Recruitment took place between March 2022 and July 2023 using these methods.

#### Facebook banner adverts

We posted short advertisements on Facebook using algorithms to target specified demographics (eg, age, gender, location and interests).

The adverts displayed a link to an external webpage hosted by Jisc Online Surveys^30^ containing a short screening questionnaire that determined eligibility. The screening questionnaire collected women’s name, age, smoking status, weeks’ gestation, email address and telephone number.

#### Social media posts

We set up accounts on different social media sites and forums (eg, Reddit, Mumsnet, Twitter) and posted links to the short screening questionnaire.

#### Participants from other studies

We also contacted participants from other studies conducted by the research group that had given consent to, and shown interest in, being involved in other research projects. Only participants where it was deemed that there was little possibility of cross contamination between ongoing projects, for example, if they were screened and found to be ineligible for an alternate study, were invited to complete the screening questionnaire for this study.

All women who completed the screening questionnaire and fulfilled the eligibility criteria were emailed a Participant Information Sheet. After 24 hours, a member of the research team made three attempts at contact to explain more about the study and offer the option of taking part in a telephone interview at a mutually convenient time.

### Interviews

Three researchers conducted the interviews (LP: female, MSc, health psychology background, non-smoker, RT: male, PhD, health psychology background, ex-smoker, SO: female, PhD, health psychology background, non-smoker). The interviewers introduced themselves as researchers from the University of Nottingham, obtained informed consent and recorded pregnancy and smoking information before commencing the interview. Interviews were audio recorded and transcribed verbatim by an external transcription service. Interviewees received a £20 shopping voucher as compensation for their time.

Interview topic guides were semi-structured and informed by the Theoretical Domains Framework^31^ and COM-B model^32^ covering the following topics: healthcare support for smoking during pregnancy, views on reducing smoking in pregnancy rather than stopping, knowledge and experience of NRT, barriers and facilitators to using NRT to reduce smoking and support/strategies for using NRT to reduce smoking in pregnancy (see online supplemental file 1). Interviews lasted 30–40 min.

### Analysis

The data were analysed using inductive thematic analysis. This approach allowed themes and patterns within data to be identified, interpreted, organised and described.^33^ Analysis was led by RT with the coding checked by a second researcher (LP) and was facilitated using NVivo 12 software.^34^ Using the method outlined by Braun and Clarke,^33 35^ the researcher who led the analysis familiarised himself with the data by reading and re-reading transcripts, systematically noting initial codes and patterns across the data. These were then collated into potential themes and subthemes, with all examples of the themes within the data gathered. Next, these themes were reviewed by RT and LP, ensuring they reflect the coded extracts and the entire data set. The themes were then further refined, with clear definitions and names for each theme given. All members of the research team provided input regarding reviewing and refining the final themes.

### Patient and public involvement

Three women from our public involvement advisory panel, all of whom had lived experience of smoking during pregnancy, were involved in the funding application and the development of participant materials (eg, recruitment adverts topic guides for interviews, participant information sheets). They were also involved in the interpretation of the data. This involved asking them to read and comment on a selection of anonymised transcripts which provided valuable insights into the importance and inclusion of potential themes and allowed us to check how the data related to their own lived experience.^36^

## Results

48 eligible women expressed an interest in taking part, from whom, we recruited 18 interviewees (10 via Facebook adverts, two from other social media posts and six from other studies). From the six women recruited from other studies, three did not meet the eligibility criteria for an NRT cessation study and three were from a carbon monoxide monitor study. We were unable to contact 30 women.

Of the 18 interviewees (mean age: 30 years), 15 reported having reduced their smoking since finding out they were pregnant, while three interviewees reported having stopped smoking. Pregnancy gestation ranged from 8 weeks to 36 weeks (mean: 20 weeks) with six interviewees reporting having smoked in a previous pregnancy. Nine interviewees had other children with eight being married, seven cohabiting and three reporting being single. Of the 18 interviewees, 10 were not actively engaged with stop smoking services, 12 had used NRT previously and nine had used NRT during their current pregnancy to assist in reducing their smoking. See table 1 for full interviewee characteristics. During analysis, we considered 18 participants provided us with adequate information power,^37^ in terms of the quality of the interview dialogue, to offer sufficient new knowledge and insights in line with the aim of the study.

**Table 1:** Interviewee demographics

Interviewee	Age	Smoked in previous pregnancies	Pre-pregnancy smoking amount (cigarettes per day)	Current smoking amount (cigarettes per day)	Recruitment method
1	21	No	5	2	Facebook
2	35	No	8	3	Facebook
3	32	Yes	15	2	Facebook
4	34	No	10	1	Facebook
5	24	Yes	20	15	Facebook
6	31	N/A	20–30	5–10	Facebook
7	25	No	50	20	Facebook
8	25	N/A	8	6	Facebook
9	32	Yes	12	4	Facebook
10	25	Yes	6	1	Facebook
11	24	Yes	5–10	1–3	Social media (Reddit)
12	35	Yes	12	8	Other study
13	37	No	12	0	Other study
14	25	No	10	0	Other Study
15	38	No	20	Less than 1	Other Study
16	32	No	25	0	Social media (Reddit)
17	31	Yes	25	6	Other study
18	30	Yes	25	10	Other study

Interviewees expressed varied views on smoking reduction in general and specifically on using NRT to reduce rather than stop smoking. These views are organised into three themes: (1) ‘views on smoking reduction’, (2) ‘views on using NRT for smoking reduction’ and (3) ‘advice and support needs’.

The findings are illustrated by extracts from participant interviews to bring transparency to the qualitative analysis. Interviewee identification numbers and whether, at the time of the interview, they had quit (Q) or reduced (R) their smoking or had used NRT to help them reduce their smoking in this pregnancy (NRT) are reported in parenthesis.

### Theme 1. Views on smoking reduction

All the women we spoke to describe making efforts to reduce or stop smoking since finding out they were pregnant. Some were cut down as an alternative to abstinence:

Well I think cutting down, if you can’t quit then cutting down would obviously be, you need to do one or the other really. I mean I cut down from 50 to 20… I don’t know if I could give up the full lot. (Int 7,R)

Others said they were cutting down with a view to quitting in the future:

…I don't feel like I physically need one every day now, I can go a day or two without one,…, hopefully I’ll completely stop and then it’s like gone forever! (Int 10, R, NRT)

Stopping smoking was seen as being particularly difficult. Most of the women had tried to stop in the past but found it either too difficult or had stopped for a while but then relapsed back into smoking:

It’s not as daunting as just completely stopping. It does feel like I’m making a better decision (cutting down) rather than just stopping completely, because before I’d literally, I would just stop buying cigarettes and then it would become a problem because I’d become agitated, so it was just becoming a massive issue. (Int 5, R)

All women spoke about the difficulties of having to cope with the symptoms of tobacco withdrawal and the feeling of having to give something up was a common theme that was discussed as to why cutting down was considered easier than complete cessation:

I know giving up is quite hard but cutting down I can kind of live with that. I think the main reason is I’m kind of you telling myself it’s OK, I can still smoke but it’s just less. So, I think the fact that I’m still smoking has given me that peace of mind (from the stress of having to quit). (Int 2, R)

However, one woman, who had stopped smoking using NRT, was very much against using a reduction approach as they felt it would be too easy for relapse to pre-pregnancy levels of smoking:

…if anything, I think it opens the pathway to temptation a bit more… You have a bad day and you're like oh sure what’s another one, what’s another one, what’s one evening of a couple more, you know? Because you're still buying them and you've still got access to them, like, one of the things that we did when we found out we were pregnant I had cigarettes in the house is I made sure that they were binned straightaway with no access for me to get to. (Int 16, Q)

#### Views on cutting down and harm reduction

Half of those interviewees who believed they had successfully reduced their smoking expressed the view that reducing the amount of cigarettes they smoked would reduce the risk of harming their unborn child:

Well obviously, if you’re cutting down, you’ve got less toxins and less carcinogens going into your body and less of it going into the baby. (Int 7, R)

However, this view was not necessarily based on any advice, it was more of an intuitive view of how to reduce the risk to the fetus, and there was some uncertainty as to the efficacy of this approach in the context of the interview:

…think I’m not exactly sure because obviously I’m not a medical professional or anything but personally I would think your baby would be at less risk… but I’m not sure whether that is true or not, if that makes sense? (Int 1, R)

There was an acknowledgement that cutting down was a compromise, and women believed that although harm was reduced, it was not eliminated:

…yeah maybe the less of the substances are going into the bloodstream and the baby maybe… (but) you still smoke, so you still poison the baby, yeah. (Int 15, R, NRT)

#### Stigma around cutting down but continuing to smoke

The stigma associated with continued smoking in pregnancy, even at a reduced amount, was identified as an important barrier for cutting down smoking rather than complete cessation. Over half of interviewees spoke about still being seen by others as a ‘pregnant smoker’ who feels they are doing something wrong. The idea of people on the outside not understanding their individual situation was expressed in descriptions of feeling judged by people who would not be aware of how difficult they were finding stopping smoking and the lack of recognition for the progress they had made in reducing their smoking:

I’ve been pregnant and smoking and I’ll still go – doesn’t look really good, does it?… Well, no one looks at how many you’re smoking a day. They just see what’s currently there which is a pregnant woman smoking. (Int 6, R, NRT)

Two thirds of interviewees reported hiding their continued, although reduced, smoking from friends and family, fearing disapproval.

My nan hates it. She absolutely hates it. She’s getting very broody obviously because first great-grandchild so she’s very strict with me and I don’t smoke when I’m at her house but as soon as I get home, I do. (Int 7, R)

They reported feeling embarrassed and guilty and did not want to be judged as possibly harming the unborn baby. Some women also reported feeling disapproval or judgement from their healthcare professionals and so were reluctant to disclose their smoking status or discuss with them that they had been unable to quit completely and so had instead reduced their smoking:

Yeah, I told my midwife I don’t smoke anymore because she’s quite judgemental. So she doesn’t actually know I still smoke. (Int 4, R, NRT)

### Theme 2. Views on using NRT for smoking reduction

Despite not having been advised to do so by any health professionals, during this pregnancy, half of interviewees had already used NRT to help reduce their smoking. There was overwhelming support among interviewees for the idea of having a recognised approach to using NRT to help women reduce their smoking when stopping completely was unobtainable:

I mean it, well hopefully it will help. I feel like if you can’t quit, and you can cut down and there’s things available to help, then why not help?… So, I’m sure if they give an option “OK we know you can’t quit right now, we know that’s not something that you can do right now, so here is something to help you cut down” I feel like that’s amazing. (Int 5, R)

Women improvised different, ad hoc, strategies to reducing their smoking such as lengthening the time between cigarettes or trying to only smoke at certain times, without any clear goal setting but could also see a way of integrating NRT to replace some of the cigarettes they smoked:

…yeah it might work to have a normal cigarette let’s say in the morning and in the evening and then during the day for example use the replacements. (Int 9, R, NRT)

One interviewee pointed out that the type of NRT most appropriate to help reduce smoking might need to be determined on an individual basis:

Mine is so habitual, it’s all about that. That’s why the inhalator works best for me. But for other people if it is purely a chemical then, you know, probably the patches would help brilliantly for them. (Int 3, R, NRT)

And that changes due to pregnancy may influence what type of NRT may be tolerated:

Personally, I have tried the gum that you can buy, but it’s the taste for me so I couldn't really have it because I was quite – early on in pregnancy I was quite sicky – so I couldn’t have the texture or the taste of it, so that went out the window. (Int 11, R, NRT)

#### Embarrassment associated with NRT

As many of the women were not actively engaged with a stop smoking service, they were having to source their own NRT and were conscious that by buying NRT in public they may be perceived by others as continuing to smoke while pregnant, even though NRT could equally be a sign of them having stopped smoking. Similar to the stigma associated with reduced smoking in pregnancy, over a quarter of interviewees described embarrassment when purchasing or using NRT while pregnant.

Being seen going to a pharmacist to purchase NRT while visibly pregnant was embarrassing for one woman, who described making her husband buy it on her behalf:

…like I made my husband carry the gum yesterday in Boots. Like I told him “I need to go and get some more gum” and like I wasn’t going to hold it. I’ll pay for it. We paid at the counter together but I don’t want to be seen even taking NRT when I’m quite clearly pregnant. (Int 4, R, NRT)

Women indicated that they would consider their choice of NRT based on how obvious it would be to others that they were using certain products:

I think if I was to open a patch out in public people would stare at me but if I had some maybe chewing gum I could kind of open the packet in my bag and kind of sneak it into my mouth because people would think it’s just normal chewing gum. (Int 2, R)

#### NRT cost

As in England, NRT is only provided at no cost to pregnant women who are in quit attempts, the expense of buying NRT for smoking reduction was seen as a barrier to this use of the treatment.

It is very expensive to do it self-funded… you don’t realise obviously say 7 or 14 day patches and you think right that’ll do me for the next 2 weeks, but you blink and then you need a new packet and then you run the risk (of running out) if you don’t have the money mid-month and especially with the cost of living happening at the moment, it’s just expensive. (Int 16, Q)

One interviewee had problems getting to the pharmacist on her day off at the weekend so had to absorb a higher cost to buy NRT from the internet:

…but I eventually gave up and just buy them on Amazon and we’ll just eat the cost, because the practicality of, like I can get Prime 1 day delivery, it’ll come out of the budget somewhere …. (Int 11, R, NRT)

#### Expectations around NRT

Over half the interviewees had previous experience of using NRT, both inside and outside of pregnancy, with varying success that may have influenced their enthusiasm for using NRT in the future. Some had previously tried different types of NRT products without successfully quitting smoking:

So, I’ve used nicotine patches. I was given them by a health professional to help – didn’t seem to work. I’ve tried the gum, that didn’t seem to work. I’ve tried to go cold turkey and then I ended up just smoking more than what I was smoking in the first place. (Int 5, R)

While others had used NRT in a successful quit attempt:

Yeah, when I quit before, probably last year, I used patches and gum. (Int 4, R, NRT)

Friends’ mixed experiences with NRT also appeared to have contributed to some participant’s mixed views about NRT. These experiences related to both NRT efficacy and product side effects:

And I know that from my friends’ experiences with like the patches for example, they haven’t really helped them cut down at all… I get like mixed responses about the patches… Some say that they’re good, and some say that they don’t really do anything. (Int 14, Q)

I mean I’ve got a certain friend and she’s got like really sensitive skin, and she told me when she put the patches on she reacted really horribly to them. (Int 2, R)

#### Safety concerns of using NRT and smoking

There were some concerns expressed about the safety of using NRT and smoking at the same time and the possibility of getting too much nicotine:

I've got mixed feelings about it because I feel like when you smoke and when you use the therapy as well you might give your body more substances, more nicotine… because you're topping it up with the patches. (Int 9, R, NRT)

One interviewee thought that there should be a way of controlling the amount of nicotine that women take in and that some NRT products may be more useful at achieving this:

The only one I could see working is maybe you know the inhalers, where you can control the intake, yeah, you can control the intake of the nicotine going into your system so if you are going to have that fag, you’re not overloading but the patches wouldn’t work… There’s no point putting on a twenty a day patch and then smoking almost twenty a day. You’ve doubled your intake. At least with you know the gum, you can spit the gum out. You can remove gum, you can remove, everything else is removable. The patches aren’t! (Int 6, R, NRT)

Two interviewees reporting feeling sick while using NRT gum and continuing to smoke and worried they may have ‘overdosed’ on nicotine:

I’ve made myself feel very sick with the gum and with the patches by trying to have a cigarette on them. And I don’t know, that would be something I’d have to actually ask a doctor as to whether I’ve just made it up – coincidence or if it is that you can have too much nicotine. (Int 3, R, NRT)

The gum tastes absolutely repulsive! And I always after having it, I felt like I needed to have a cigarette and then I’d actually often find that I almost – I don’t know if you could call it this, but I overdosed on nicotine and made myself feel very sick. (Int 15, R, NRT)

### Theme 3. Advice and support needs

When considering what advice or support from health professionals would be helpful if NRT for smoking reduction in pregnancy were presented as a treatment option, women felt a tailored, encouraging approach that was offered early in pregnancy would be helpful.

Women reported the importance of adopting a tailored approach to supporting pregnant women use NRT to help reduce their smoking when abstinence was unachievable:

I would say every woman is different and everybody smokes different ways. Some smoke more than each other because all women are different… (how) slowly you cut down– depends on the person or the lady, how long it takes them. (Int 1, R)

It was suggested that women should have individualised reduction targets that could be monitored using an app to record the number of cigarettes smoked or by expired carbon monoxide measurements:

I don’t know if (stop smoking services) could come to a compromise in terms of if you're cutting down but you're still going to be smoking only have ‘x’ amount of cigarettes or however they want to measure it in terms of, I don’t know, like carbon monoxide detects or anything like that be helpful, you have to be under a certain threshold by sort of that midway point. (Int 16, Q)

There currently seemed little encouragement for women that had managed to reduce their smoking with NRT. This was highlighted by interviewees as an area that would need to be improved:

[A] bit of validation that actually (motivates you). it’s a hard thing you’re trying to do. I think at the moment it’s very much focused on “You must quit and if you don’t, this is what you’re doing to the baby”. Rather than that, flip it so you get a bit more validation of actually ’This is a really difficult thing you’re doing, and you have actually cut down by quite a considerable amount’. (Int 18, R, NRT)

And that if women were able to receive support cutting down their smoking with NRT, it should be seen as an equally acceptable ‘treatment choice’, and they should receive equal support and encouragement:

…if somebody says “Look, I don’t think I’m going to be able to stop but I am more than happy and willing to try and cut down with the right support and resources and things” then I feel like that should be encouraged just as much as being encouraged to stop. (Int 14, Q)

When women were asked when the best time would be to offer NRT to reduce their smoking, most recommended that it should be offered early on in pregnancy to limit the harm to the fetus:

I think early, it’s best to start early in pregnancy so you can, sort of, give your baby the best chance, yeah. And to give yourself the time because if you start late in pregnancy, I find there is no point in starting. (Int 13, Q)

## Discussion

This study reports the exploration of the acceptability, in reducing the number of daily cigarettes smoked in pregnancy, rather than for stopping smoking completely and the potential role NRT may have in this process. The analysis identified an appreciation for a ‘cutting down’ approach to smoking harm reduction while highlighting associated difficulties. Half of interviewees reported using NRT to help them reduce their smoking in this current pregnancy, while the perceived stigma associated with purchasing or using NRT were seen as problematic. When presented with the suggestion that NRT could be used to help women reduce their smoking, issues associated with using NRT in this way were discussed.

A key strength of our study is that we are unaware of any others that have qualitatively explored the views of pregnant women on whether NRT should, or could, be used to help reduce the number of cigarettes smoked when complete cessation was not likely, and the kind of support needed for this approach. Online recruitment using Facebook adverts and social media posts allowed researchers to identify and interview women from across the UK who might be disengaged from SSS (ie, those who were unable or unwilling to stop smoking).^38^

The main study limitation was the reliance on telephone, rather than face-to-face, interviews. While it is more difficult to develop rapport with interviewees over the phone, this approach is known to have advantages when discussing topics of a potentially sensitive nature.^39^ Furthermore, the use of social media recruitment, while advantageous in terms of geographical reach, may have excluded representation from those who do not use social media regularly or use platforms other than Facebook.^40^

For interviewees in this study reducing their smoking seemed to be an intuitively adopted behaviour and is congruent with the idea that particular health behaviours such as smoking, drinking alcohol and healthy eating may change as a result of pregnancy and without intervention from health professionals.^41^ Interviewees in this study understood the opportunities afforded by adopting a cutting down approach, not only because abrupt stopping was seen as particularly difficult but also because smoking reduction was seen as a way of reducing the harms related to continuing to smoke at pre-pregnancy levels. While most pregnant smokers recognise the risks to their unborn child,^42^ the findings of this study echo the sentiments expressed in other qualitative work, in that, while quitting smoking was judged to be the ideal, cutting down was seen as an important strategy in reducing the harm to the unborn fetus that should be considered.^28^

This study identified different strategies employed by women to reduce their smoking such as increasing the time between cigarettes or only smoking at certain times of the day which have been shown to be effective means of smoking reduction^43 44^; however, there seemed to be a lack of clear goal setting, which is an important aspect in behavioural changes.^45^ Having access to professional support that could assist women in distinct, realistic, goal setting and clear feedback may increase successful reduction.^46^ The lack of praise women received from healthcare professionals for any cigarette reduction was a concern raised by our Patient and Public Involvement and Engagement (PPIE) group when they reviewed interview transcripts and assisted in initial coding. They felt that in general these women were not receiving adequate support which could be a reflection of the current focus on cessation above harm reduction.

One difficulty with this approach was the stigma surrounding continued smoking. Women seemed concerned that other people would judge them negatively if they were seen buying or using NRT without understanding that they had worked hard to reduce the amount they smoked. Stigma around smoking during pregnancy has long been recognised and is considered to be less to do with the level of risk to the fetus and more of a moral judgement.^47^ The stigmatisation of pregnant women who smoke may lead to women hiding their smoking and a hesitancy to engage with professional smoking cessation support.^48 49^

We sought women who had experience of smoking in pregnancy but did not seek those who were trying to stop or to cut down smoking at all. Nevertheless, half of the women enrolled in this study were using NRT to cut down their smoking, and over half were not actively engaged with stop smoking services. This suggests that using NRT to cut down daily smoking may already be a widely adopted practice. It was unclear why the women were not using stop smoking services. A possible explanation could be that cessation services only work with women who are committed to trying to quit, and it has been shown that stop smoking practitioners (SSPs) hold particularly negative views towards using NRT for cutting down smoking in pregnancy and would only ever advise NRT use and concurrent smoking for anything but the briefest of smoking lapses during a quit attempt.^50^ Women who felt that they were unable or not willing to commit to a stop smoking attempt would find their access to free NRT products from stop smoking services restricted and may have resulted in women buying their own NRT or nicotine products themselves. Buying their own NRT without the associated support and education provided by healthcare professionals may lead to women being less informed around what to expect in terms of possible side effects, such as nausea, which may impact better tolerance and adherence to different NRT products.^51^ The cost of buying NRT to support cutting down their smoking alongside the potential for embarrassment reported by some women by buying NRT in public was seen as a barrier to this approach. Only organisational changes that would allow the prescription of NRT to support smoking reduction in pregnancy, as it is in the general population of smokers who are unable to quit, would reduce the burden on an already socially and economically disadvantaged population.^52^

Stigma was also cited as a barrier by the interviewees in our study when purchasing or using NRT to help them reduce their smoking. This finding emphasises other work that pregnant smokers who feel stigmatised may be particularly attracted to products that can be used discreetly^53^ such as patches that can easily be hidden under clothing or nicotine gum that looks like an ordinary product. It was reported that purchasing nicotine products over the counter offered more opportunities for them to feel judged by others. Unless women were able to procure NRT to cut down their smoking on prescription or directly from a stop smoking service, perhaps using an online delivery service may help alleviate these concerns; however, this may add an additional cost to these products.

Some women reported being concerned about getting too much nicotine from using NRT and smoking at the same time; however, a recent review and meta-analysis should provide reassurance that using NRT alongside smoking in the context of smoking reduction is unlikely to result in dangerous levels of nicotine exposure.^54^ This may be because most smokers are able to self-titrate their nicotine intake, through smoking and NRT, to maintain plasma nicotine levels without adverse physical or subjective effects.^55^

Similar qualitative work is needed with health professionals to assess their views on this topic. Given that stop smoking practitioners have previously reported not feeling comfortable promoting a harm reduction approach to pregnant women, it was not compatible with their aims of promoting a smoke-free pregnancy and healthy baby^50^ and that stopping smoking completely is the only way of ensuring that the unborn baby is not at risk from smoking harms.^13^ Health professionals might need to consider how they balance communicating the risks of reduced but continued exposure to tobacco smoke and the use of NRT to promote smoking reduction as a possible treatment option without undermining cessation as the aim of a stop smoking service. This is particularly important when pregnant women are often confused about the need for the safety of NRT and clear, consistent messages from healthcare professionals.^51^

There was a distinct preference for offering NRT to support smoking reduction as early as possible in pregnancy in an attempt to minimise the effects of smoking. There is a suggestion that women may be more motivated to quit earlier on in pregnancy,^14^ so it seems reasonable to suggest that women may also be more motivated to reduce their smoking during this time frame. As midwives play a particularly pivotal role in providing SSS to pregnant women,^56^ any steps to reduce stigma around reduced smoking, procurement of NRT and the provision of empathetic support would require meaningful co-development involving both midwives and pregnant women.^47^

## Conclusion

Using NRT to help women who are unable to stop smoking, reduce their smoking, is potentially acceptable to pregnant women. This study found women were already using NRT alongside ad hoc strategies to reduce their smoking. There are barriers to adopting this approach, associated with access to NRT and current attitudes towards smoking during pregnancy. Further research involving a structured smoking reduction plan, alongside the concurrent use of NRT and proper follow-up care, is needed to evaluate this approach.

## supplementary material

10.1136/bmjopen-2024-085945 online supplemental file 1

## Data Availability

Data are available upon reasonable request.
