# Supplementary material for: Using nicotine replacement therapy for smoking reduction in pregnancy: a qualitative study of pregnant women in the UK who smoke
Source: BMJ Open. 2024 Aug 30;14(8):e085945. doi: 10.1136/bmjopen-2024-085945 (PMC11407210; doi:10.1136/bmjopen-2024-085945)
Supplement: online supplemental file 1 [file bmjopen-14-8-s001.pdf]

Topic guide: Using NRT for smoking reduction in pregnancy

Introduction: Aim, to create an appropriate atmosphere

- Name of the interviewer, and affiliation
- Purpose of the study
- Funding – this study is being funded by the National Institute for Health research
- Ensure the PIS has been read and understood
- Confirm participant has given their consent to take part in the study
- Reiterate that information collected as part of the study will be confidential, and any quotes used from the interview will be anonymous so that they will not be able to be linked back to them as an individual
- Interview will last approximately 30-40 minutes
- The interview will be audio recorded so that the interviewer can fully engage in the interview
- Emphasise there are no right or wrong answers and that we are interested what she thinks and feels about the subject we are going to talk about

Warm up questions: ***Aim, context about smoking and make women feel comfortable***
Smoking and pregnancy can be a difficult topic for some people to discuss. Some women carry on smoking during pregnancy, either because they want to or they try to stop and can't. Others try to stop and manage this, and a group of women try very hard to smoke as little as possible.

The research team realise it's very hard to stop smoking and we can only develop new ways of helping pregnant women to stop smoking if we know more about how and why women smoke. There is no judgment of you being made, and your valuable input and honesty could help others in the same situation.

The team / I'm really grateful for you giving up your time. We really want to hear about your smoking and what you think about this and we aren't looking for any particular answers to our questions.

**Question**	***Prompts***
Can you tell me how long you have been smoking?	
How do you feel about your smoking in general?	
Can you tell me how you feel about your smoking now that you are pregnant? Do you feel different?	

<u>Healthcare support for smoking during pregnancy:</u> ***Aim, to identity women's interactions with healthcare professionals when smoking during pregnancy and what support they were offered (if any)***

**Question**	***Prompts***
Have you had any conversations with any healthcare professionals (e.g. Midwives or GP) about your smoking during your pregnancy?	What advice did they give you?<br>• How easy or difficult was it to follow this advice?<br>• Is there anything you would have liked them to discuss that they didn't mention or advise on?
Have you been given any advice about smoking in pregnancy by anyone else? <u>(such as family, friends?)</u>	• *(if applicable)* Did this advice differ from advice from a health professional? How?<br>• How did this advice make you feel?

<u>Views on reducing smoking in pregnancy</u>: Aim, identify their views on reducing smoking during pregnancy, for women who can't stop.

As you may have found, NHS practitioners generally advise pregnant women to stop smoking ***completely*** on a quit date, and after this to not smoke at all. Some women find this hard and have said that, if they can't stop completely, they would rather try to cut down.

**Question**	***Prompts***
What do you think about the idea of cutting down smoking in pregnancy rather than stopping completely, for women who have not been able to stop smoking completely?	• What do you think would be the advantages of this approach, if any?<br>• What do you think would be the disadvantages of this approach, if any?
If you couldn't stop smoking and wanted to try cutting down instead…… what might help you with this?	
If you couldn't stop smoking and wanted to try cutting down instead…… what might make this harder for you, get in the way of you doing this?	

Do you have any personal experience of trying to cut down your smoking when pregnant?	If yes:<br>• How did you find this?<br>• Were there any barriers (e.g. things that made it difficult) to cut down?<br>• Were there any facilitators (e.g. things that helped/made it easier?) to cut down?<br><br>If no:<br>• Is this something you might consider doing?<br>• Does it sound like a good idea?- why?<br>• If not, why not?

Knowledge and experience of NRT: ***Aim, identify any NRT use during pregnancy, attitudes towards NRT, and use during pregnancy.***

Next, we want to ask about using nicotine replacement therapy when pregnant.

Nicotine replacement therapy (or NRT) NRT is a medication that provides you with a low level of nicotine, without other poisonous chemicals present in tobacco smoke. It can help reduce unpleasant withdrawal effects which may occur when you stop smoking.

NRT can be taken as nicotine patches, gum, lozenges, inhalators or mouth sprays.

**Question**	***Prompts***
Have you heard about NRT before?	
Have you ever used NRT (either before or during pregnancy)?	If yes:<br>• When did you use NRT? (e.g. outside of pregnancy, during pregnancy)<br>• What types of NRT did you use?<br>• How did you find it? (e.g. how effective, any side effects)<br><br>If no:<br>• Are there any reasons why you haven't tried or used NRT?

<u>Barriers and facilitators to using NRT to reduce smoking</u>: Aim, identify barriers and facilitators to using NRT to cut down smoking during pregnancy

One idea that researchers are thinking about is offering NRT to women who are unable to quit smoking in pregnancy completely, to instead help them to reduce their smoking until their baby is born.

**Question**	***Prompts***
What do you think about the idea of offering NRT to women who are not able to quit smoking completely in pregnancy, to instead cut down their smoking?	• Do you think there are there any positives to this approach?<br>• Do you think there are any negatives to this approach?
What are your views on the safety of using NRT to cut down smoking, rather than for stopping smoking?	
Can you think of any barriers (e.g. things that might make using NRT to cut down smoking more difficult)	
Can you think of any facilitators (e.g. things that might make using NRT to cut down smoking easier)	
What types of NRT, for example, patches, gum, inhalators, do you think would be most appropriate to use alongside reducing smoking?	Why?
Have you ever used NRT to cut down the number of cigarettes you smoked while you were pregnant?<br><br>NOTE: may come up elsewhere in interview if they have used NRT and *unintentionally* reduced their smoking as a result.	If yes:<br>• Why did you try this approach?<br>• How did you find it?<br>• Did you find it helpful? Why?<br>• How did you decide what type of NRT to use?<br>• How did you decide what dose of NRT to use?<br>• How did/do you decide when to use NRT, and when to smoke?<br>• To what extent did/does using NRT satisfy your cravings for cigarettes, if at all?<br>• Were there any difficulties you experienced?<br>• What did other people (e.g. family, friends) think about you using NRT to cut down rather than quit?<br><br>If no:<br>• Is this something you might consider doing?

	• Does it sound like a good idea?- why?<br>• If not, why not?<br>• Can you think of any issues you or other people may have in using NRT to cut down smoking
If you were to be offered NRT to help you cut down smoking in pregnancy, when would be the best time for this to happen?	Prompts for timings/examples of different times:<br>• Early in pregnancy?<br>• Later in pregnancy?<br>• After you have had other support to quit pregnancy?<br>• After an unsuccessful quit attempt?<br>• Why?
Do you know anyone else that has used NRT to help them cut down smoking in pregnancy?	• Did they think this approach was helpful?<br>• Did they say what was easier/more difficult about cutting down instead?

<u>Support/strategies for using NRT to reduce smoking in pregnancy</u>: Aim, identify what support women feel would be helpful to initiate and maintain NRT use in pregnancy, how this support should be obtained.

**Question**	***Prompts***
If a stop smoking advisor, midwife or doctor were to suggest that because you were finding it difficult to stop smoking that you might use NRT to cut down instead- what information or support would you like to receive to help you feel confident in this approach	• Can you envisage(/imagine) how this would make you feel?<br>• Is there any information you would like to be given about cutting down?<br>• Why?<br>• Is there any support that you think would be useful to help you cut down?<br>• Why?

AT THE END OF THE INTERVIEW, ENSURE YOU COLLECT ADDRESS FOR SENDING VOUCHERS AND THANK PARTICIPANT FOR THEIR TIME.
