# Supplementary material for: Using nicotine replacement therapy for smoking reduction in pregnancy: a qualitative study of pregnant women in the UK who smoke
Source: BMJ Open. 2024 Aug 30;14(8):e085945. doi: 10.1136/bmjopen-2024-085945 (PMC11407210; doi:10.1136/bmjopen-2024-085945)
Supplement: Supplementary file 2 [file bmjopen-2024-085945.reviewer_comments.pdf]

PEER REVIEW HISTORY

BMJ Open publishes all reviews undertaken for accepted manuscripts. Reviewers are asked to complete a checklist review form and are provided with free text boxes to elaborate on their assessment. These free text comments are reproduced below.

ARTICLE DETAILS

**TITLE (PROVISIONAL)**	Using NRT for smoking reduction in pregnancy: A qualitative study of pregnant women in the UK who smoke
**AUTHORS**	Thomson, Ross; Phillips, Lucy; Orton, Sophie; Naughton, Felix; Coleman, Tim

VERSION 1 - REVIEW

**REVIEWER NAME**	***Bar-Zeev, Yael***
**REVIEWER AFFILIATION**	Hebrew University of Jerusalem Faculty of Medicine, Braun School of Public Health and Community Medicine
**REVIEWER CONFLICT OF INTEREST**	YBZ has received honoraria for a talk and travel expenses from manufacturers of smoking cessation medications, including NRT (Pfizer, Novartis NCH, and GSK consumer health) in the past (2012-July 2019).
**DATE REVIEW RETURNED**	17-Apr-2024

**GENERAL COMMENTS**	This is an important qualitative study on the acceptability of using nicotine replacement therapy for smoking reduction during pregnancy as a harm reduction approach. The article is clear and I enjoyed reading it. I only have some minor comments:<br>Abstract<br>1. Please provide full name of NRT for first use in abstract<br>Introduction<br>1. Although the authors mention that there are concerns regarding use of NRT in pregnancy they only mention that these may prove unsubstantiated. It would aid the readers to include a bit more context and state that exposure to nicotine in animal studies has been shown to impair fetal brain and lung development, and that currently nicotine is classified as a category D for pregnancy use by the US FDA and by the Australian TGA (https://www.drugs.com/pregnancy/nicotine.html)<br>Methods<br>2. For the inclusion criteria – please clarify if smoking included all tobacco or nicotine products or only combustible cigarettes? Did you recruit also women who only used electronic cigarettes?<br>3. Can you add how many members are in your public involvement advisory panel? I assume they are all female as you mention that they had lived experience of smoking in pregnancy, but maybe better to state that explicitly.<br>Results<br>4. You mention that 18 were recruited out of 48 that expressed interest. Why were the other 30 not recruited?<br>5. How did you decide to stop interviews after 18 participants?<br>Discussion<br>6. Please consider adding a bit regarding the fact that discussions with health professionals around this as a strategy will need to balance the fact that this is a harm reduction approach (and will not eliminate all the risk), continued risk from exposure to cigarettes but

	also to nicotine from the NRT (albeit less than continuing to smoke), including fear that this may prevent/dissuade some from completely quitting (acknowledging it's a difficult balance). Similar work needs to be done with health professionals to assess their views on this topic.

**REVIEWER NAME**	***O'Donnell, Rachel***
**REVIEWER AFFILIATION**	University of Stirling, Institute for Social Marketing and Health
**REVIEWER CONFLICT OF INTEREST**	I have no competing interests to declare.
**DATE REVIEW RETURNED**	30-May-2024

**GENERAL COMMENTS**	Thank you for the opportunity to review your paper 'Using NRT for smoking reduction in pregnancy: A qualitative study of pregnant women who smoke'. The paper is very well written and it was a pleasure to read. This is an important topic area, with little previous research conducted, and a need for evidence to inform future clinical practice. I therefore recommend the paper is accepted for publication subject to minor revisions, outlined below, which I hope you find helpful:<br>1. The introduction is very well written and provides the reader with a clear, succinct overview of the background/context to the study. It is noted that babies born to women smoking fewer cigarettes per day are heavier than those born to women smoking a greater number of cigarettes per day. I wondered if concerns about having a heavier baby due to smoking less were shared during the interviews by any of the women involved in the study? This could be a potential barrier to use of NRT during pregnancy for some women, and it would be interesting to note whether issue was raised during interviews.<br>2. Methods - Participants included individuals who had been involved in other studies conducted by the team. Could you clarify what proportion/how many of the overall sample fall into this category, whether these previous studies involved use of NRT, and if so, how this (or any other aspect of their prior experience of taking part in studies conducted by the team) might impact on findings and conclusions drawn?<br>Analysis - The authors refer to Braun and Clarke's 2006 paper (reference 31) - using thematic analysis in psychology. Are there any newer B&C references that could be used as their thinking on approaches to analysis and different types of thematic analysis has evolved quite significantly since this paper was published.<br>3. Patient and Public involvement statement - how many members of the panel were involved in shaping this work? Could you add a sentence or two to briefly describe HOW their involvement shaped the work. The sentences on PPI that appear in the analysis section allude to this, but would be better placed in the PPI section of the methods.<br>4. How were the 18 interviewees selected from the pool of 43 eligible women?<br>5. Interviewee demographics - did you collect any data on socio-economic status?<br>6. Results/Discussion - there are a few quotes that suggest the importance of women knowing how to use NRT effectively/understanding initial side effects of NRT use. For example, on page 12 nausea associated with initial product use is mentioned, and challenges associated with getting used to the taste of NRT products are mentioned elsewhere. Was any information

	provided on possible side effects and common experiences of initial NRT use? Perhaps this might help to increase the likelihood of continued use beyond the first few days. This was suggested as important by participants in recent pilot studies we conducted on use of NRT to create a smoke-free home (in a broader population, not specifically pregnant women) and there are some parallels which may be of interest (including on stigma too):<br>Howell R et al (2023). Use of nicotine replacement therapy to reduce children's exposure to second-hand smoke in the home: a qualitative pilot study involving local community pharmacies. BMC Public Health. 2023 Dec 20;23(1):2545.<br>Lewis G et al. (2020). Stigma and Smoking in the Home: Parents' Accounts of Using Nicotine Replacement Therapy to Protect Their Children from Second-Hand Smoke. Int J Environ Res Public Health. 2020 Jun 17;17(12):4345.<br><br>7. Discussion: You note that access to professional support may be important to support women to successfully reduce smoking using NRT. Could you expand on this by adding who might be best placed to provide such support? You note that there may be embarrassment with buying NRT over the counter suggesting that community pharmacies may not be best placed to provide such support (the paper outlined above notes other challenges with involving community pharmacy staff, for example, given the need for a face to face discussion in the pharmacy about smoking behaviours). So who might be better placed to provide support and follow up care?

VERSION 1 – AUTHOR RESPONSE

Response to reviewer 1.

Abstract
1. Please provide full name of NRT for first use in abstract

Response *NRT is now defined at first use in the abstract*

Introduction
1. Although the authors mention that there are concerns regarding use of NRT in pregnancy they only mention that these may prove unsubstantiated. It would aid the readers to include a bit more context and state that exposure to nicotine in animal studies has been shown to impair fetal brain and lung development, and that currently nicotine is classified as a category D for pregnancy use by the US FDA and by the Australian TGA (https://www.drugs.com/pregnancy/nicotine.html)

Response: We thank the reviewer for this comment. *A section added to the third paragraph of the introduction noting the results of animal studies and the classification of NRT use in pregnancy in The U.S and Australia*.

"Pregnant women are not recommended NRT for reducing smoking due to safety concerns. Many animal studies demonstrate that nicotine could be harmful to the developing fetus[20], and, in the US and Australia, nicotine is classified as potentially a risk for use in pregnancy. [21] Whilst it would not be logical to advocate nicotine use in pregnant women who do not smoke, systematic reviews suggest using NRT instead of smoking is protective not harmful to the fetus[22 23]. . .

Methods

2. For the inclusion criteria – please clarify if smoking included all tobacco or nicotine products or only combustible cigarettes? Did you recruit also women who only used electronic cigarettes?

Response *We have clarified smoking to mean 'cigarette smoking' but that we also included women who either combined or replaced cigarette smoking during this pregnancy with other nicotine containing products.*

3. Can you add how many members are in your public involvement advisory panel? I assume they are all female as you mention that they had lived experience of smoking in pregnancy, but maybe better to state that explicitly.

Response *We agree that this would aid clarity to state explicitly. "**Three women** from our public involvement advisory panel. . . " added to the PPI section*

Results

4. You mention that 18 were recruited out of 48 that expressed interest. Why were the other 30 not recruited?

Response We were unable to contact/had no response from the 30 participants we did not recruit. We have added this information to the results section.

Page 5 "We were unable to contact 30 women."

5. How did you decide to stop interviews after 18 participants?

Response We thank the reviewer for highlighting this important aspect of our analysis. We have now added the statement *"During analysis we considered 18 participants provided us with adequate information power (Malterud et al 2016), in terms of the quality of the interview dialogue, to offer sufficient new knowledge and insights in line with the aim of the study"* to the results section.

Discussion

6. Please consider adding a bit regarding the fact that discussions with health professionals around this as a strategy will need to balance the fact that this is a harm reduction approach (and will not eliminate all the risk), continued risk from exposure to cigarettes but also to nicotine from the NRT (albeit less than continuing to smoke), including fear that this may prevent/dissuade some from completely quitting (acknowledging it's a difficult balance). Similar work needs to be done with health professionals to assess their views on this topic.

Response We agree with the reviewer that seeking the views of health professionals is important in future research. We have added a paragraph to the discussion (page 14) that suggests qualitative work with health professionals and the balance between promoting harm reduction without undermining complete cessation:

Similar qualitative work is needed with health professionals to assess their views on this topic. Given stop smoking practitioners have previously reported not feeling comfortable promoting a harm reduction approach to pregnant women because it was not compatible with their aims of promoting a smoke free pregnancy and healthy baby[50] and that stopping smoking completely is the only way of ensuring that the unborn baby is not at risk from smoking harms.[13] Health professional might need to consider how they balance communicating the risks of reduced but continued exposure to tobacco smoke and the use of NRT to promote smoking reduction as a possible treatment option without undermining cessation as the aim of a stop smoking service. This is particularly important when pregnant women are often confused about the safety of NRT and clear, consistent messages from health care professionals is needed.[51]

Response to reviewer 2

1. The introduction is very well written and provides the reader with a clear, succinct overview of the background/context to the study. It is noted that babies born to women smoking fewer cigarettes per day are heavier than those born to women smoking a greater number of cigarettes per day. I wondered if concerns about having a heavier baby due to smoking less were shared during the interviews by any of the women involved in the study? This could be a potential barrier to use of NRT during pregnancy for some women, and it would be interesting to note whether issue was raised during interviews.

Response: We thank the reviewer for this important insight, and recognise that historically women have raised concerns about giving birth to larger babies but this issue was not raised by any of the women in this study so did not warrant inclusion in this paper.

2. Methods - Participants included individuals who had been involved in other studies conducted by the team. Could you clarify what proportion/how many of the overall sample fall into this category, whether these previous studies involved use of NRT, and if so, how this (or any other aspect of their prior experience of taking part in studies conducted by the team) might impact on findings and conclusions drawn?

Response: We agree that this would be useful information to include in the methods and results. There were 6 women from other studies as noted in table 1 and in the results section.

We have now included a further breakdown of those participants in the results section:

From the 6 women recruited from other studies, 3 did not meet the eligibility criteria for a NRT cessation study and 3 were from a carbon monoxide monitor.

As already noted in the recruitment section (participants from other studies) *"Only participants where it was deemed that there was little possibility of cross contamination between ongoing projects, for example, if they were screened and found to be ineligible for an alternate study, were invited to complete the screening questionnaire for this study"*.

3. Analysis - The authors refer to Braun and Clarke's 2006 paper (reference 31) - using thematic analysis in psychology. Are there any newer B&C references that could be used as their thinking on approaches to analysis and different types of thematic analysis has evolved quite significantly since this paper was published.

Response: We thank the reviewer for this recommendation and agree with their suggestion. A further, more recent reference has been added (reference 33)

4. Patient and Public involvement statement - how many members of the panel were involved in shaping this work? Could you add a sentence or two to briefly describe HOW their involvement shaped the work. The sentences on PPI that appear in the analysis section allude to this, but would be better placed in the PPI section of the methods.

Response: We have now noted that 3 members of our panel were involved and moved and elaborated on the sentences in the analysis section, to the PPI section

5. How were the 18 interviewees selected from the pool of 43 eligible women?

Response We were not able to contact/received no response from the 30 women who were identified but not interviewed for the study. We have included this information in the results section: "We were unable to contact 30 women." Page 5

6. Interviewee demographics - did you collect any data on socio-economic status?

Response: We thank the reviewer for this question. Unfortunately, we did not on this occasion collect this information but given your comment we will endeavour to do so in future qualitative work.

7. Results/Discussion - there are a few quotes that suggest the importance of women knowing how to use NRT effectively/understanding initial side effects of NRT use. For example, on page 12 nausea associated with initial product use is mentioned, and challenges associated with getting used to the taste of NRT products are mentioned elsewhere. Was any information provided on possible side effects and common experiences of initial NRT use? Perhaps this might help to increase the likelihood of continued use beyond the first few days. This was suggested as important by participants in recent pilot studies we conducted on use of NRT to create a smoke-free home (in a broader population, not specifically pregnant women) and there are some parallels which may be of interest (including on stigma too):

Howell R et al (2023). Use of nicotine replacement therapy to reduce children's exposure to second-hand smoke in the home: a qualitative pilot study involving local community pharmacies. BMC Public Health. 2023 Dec 20;23(1):2545.

Lewis G et al. (2020). Stigma and Smoking in the Home: Parents' Accounts of Using Nicotine Replacement Therapy to Protect Their Children from Second-Hand Smoke. Int J Environ Res Public Health. 2020 Jun 17;17(12):4345.

Response: Thank you for this comment and papers. We did not specifically ask about information given around directions of use/side effects and this did not come up organically during the interviews. We agree that providing women with better information around what to expect when using NRT may encourage better tolerance and adherence and we are currently conducting a clinical trial that includes enhanced NRT information. A sentence has been added to page 13 in the discussion to reflect this.

"Buying their own NRT without the associated support and education provided by healthcare professional may lead to women being less informed around what to expect in terms of possible side effects, such as nausea, which may impact better tolerance and adherence to different NRT products.[51]"

8. Discussion: You note that access to professional support may be important to support women to successfully reduce smoking using NRT. Could you expand on this by adding who might be best placed to provide such support? You note that there may be embarrassment with buying NRT over the counter suggesting that community pharmacies may not be best placed to provide such support (the paper outlined above notes other challenges with involving community pharmacy staff, for example, given the need for a face to face discussion in the pharmacy about smoking behaviours). So who might be better placed to provide support and follow up care?

Response:

We thank the reviewer for their considered comments on the implications of our findings and agree that including this in the discussion would strengthen the paper. We have added a suggestion in the final paragraph on page 14 that midwives might be best placed to provide and design any new support package:

“As midwives play a particularly pivotal role in providing stop smoking support to pregnant women,[56] any steps to reduce stigma around reduced smoking, procurement of NRT and the provision of empathetic support would require meaningful co-development involving both midwives and pregnant women.[47]”

VERSION 2 – REVIEW

**REVIEWER NAME**	***Bar-Zeev, Yael***
**REVIEWER AFFILIATION**	Hebrew University of Jerusalem Faculty of Medicine, Braun School of Public Health and Community Medicine
**REVIEWER CONFLICT OF INTEREST**	I have no competing interests
**DATE REVIEW RETURNED**	20-Jul-2024

**GENERAL COMMENTS**	The authors have addressed all of my previous comments. I have only one minor comment - since you removed the details regarding relationship status and other children from Table 1 (due to the editor's request), you might add a sentence in the beginning of the results of these characteristics in general for the sample.

**REVIEWER NAME**	***O'Donnell, Rachel***
**REVIEWER AFFILIATION**	University of Stirling, Institute for Social Marketing and Health
**REVIEWER CONFLICT OF INTEREST**	No competing interests.
**DATE REVIEW RETURNED**	23-Jul-2024

**GENERAL COMMENTS**	The revisions made further enhance the paper, which includes important learning and recommendations for future research. In my view, this paper should be accepted as is, and I look forward to seeing it published.

VERSION 2 – AUTHOR RESPONSE

In response to reviewer 1, we have included a sentence in the results section that summarises the 'relationship status' and 'other children' information that was previously removed from the included table as follows:
"Nine interviewees had other children with eight being married, 7 cohabiting and 3 reporting being single."
